# Underwater Drone Architecture for Marine Digital Twin: Lessons Learned from SUSHI DROP Project †

**DOI:** 10.3390/s22030744

**Published:** 2022-01-19

**Authors:** Alessandro Lambertini, Massimiliano Menghini, Jacopo Cimini, Angelo Odetti, Gabriele Bruzzone, Marco Bibuli, Emanuele Mandanici, Luca Vittuari, Paolo Castaldi, Massimo Caccia, Luca De Marchi

**Affiliations:** 1 Department of Civil, Chemical, Environmental, and Materials Engineering (DICAM), University of Bologna, 40136 Bologna, Italy; alessandro.lambertini@unibo.it (A.L.); emanuele.mandanici@unibo.it (E.M.); luca.vittuari@unibo.it (L.V.); 2 Department of Electrical, Electronic and Information Engineering (DEI) “Guglielmo Marconi”, University of Bologna, 40136 Bologna, Italy; massimilian.menghin3@unibo.it (M.M.); paolo.castaldi@unibo.it (P.C.); 3 Department of Biological, Geological, and Environmental Sciences (BIGEA), University of Bologna, 40126 Bologna, Italy; jacopo.cimini2@unibo.it; 4 Italian National Research Council—Institute of Marine Engineering (CNR—INM), 16149 Genoa, Italy; angelo.odetti@cnr.it (A.O.); gabriele.bruzzone@cnr.it (G.B.); marco.bibuli@cnr.it (M.B.); massimo.caccia@cnr.it (M.C.)

**Keywords:** UUV, ROV, AUV, surveying, monitoring, marine, digital twin

## Abstract

The ability to observe the world has seen significant developments in the last few decades, alongside the techniques and methodologies to derive accurate digital replicas of observed environments. Underwater ecosystems present greater challenges and remain largely unexplored, but the need for reliable and up-to-date information motivated the birth of the Interreg Italy–Croatia SUSHI DROP Project (SUstainable fiSHeries wIth DROnes data Processing). The aim of the project is to map ecosystems for sustainable fishing and to achieve this goal a prototype of an Unmanned Underwater Vehicle (UUV), named Blucy, has been designed and developed. Blucy was deployed during project missions for surveying the benthic zone in deep waters of the Adriatic Sea with non-invasive techniques compared to the use of trawl nets. This article describes the strategies followed, the instruments applied and the challenges to be overcome to obtain an accurately georeferenced underwater survey with the goal of creating a marine digital twin.

## 1. Introduction

In the age of digitization of the existing world, the term Digital Twin (DT) was first published in 2010 within the NASA roadmap [1] defining it as an ultra-realistic, multi-scale, multi-physics simulation of a system to mirror its evolution. In the NASA document, the term DT was primarily used to identify the digital version of a vehicle designed for exploration, but later the same term was applied to several areas including: manufacturing [2,3,4], decision support systems [5], education [6], healthcare [7], climate [8], water management [9], sustainability [10], farming [11], urban planning [12], risk assessment [13] and also marine elements [14].

In recent decades, the number of instruments that allow us to observe the world at different scales and with different sensors that can capture fundamental aspects and contribute to the creation of the respective DTs has increased in parallel. Some particularly versatile platforms have seen considerable success both commercially and in the world of surveying for scientific research: drones. The possibility of carrying out a survey at a safe distance using a vehicle without an operator on board and with reduced procedures and costs compared to the past has been particularly attractive and has allowed the development of a large number of Unmanned Aerial Vehicles (UAV). Similarly, other specialized usage scenarios have developed by implementing Unmanned Surface Vehicles (USV) and Unmanned Underwater Vehicles (UUV).

Among the various projects underway for the development of DT, it is worth mentioning the recent initiative of the European Commission: Destination Earth (DestinE) [8]. The main objective of the project is the development of a digital model of the entire Earth system with high precision and resolution that can allow us to monitor natural and anthropogenic phenomena in order to support sustainable development. These systems include: land, marine, atmosphere and biosphere. Among the areas of study, the marine one is certainly fundamental for life on Earth and at the same time complex to investigate. Water covers more than 70 percent of the Earth’s surface and in particular the oceans conserve almost all the available water.

Compared to the exploration of terrestrial or aerial systems, the study of submerged environments involves greater challenges: economic, logistical and security. Exploration costs are particularly high because of the vehicles, instruments and personnel required; it is not possible to rely on permanent infrastructures that are available only in small numbers and finally the particularly harsh and hostile environmental conditions pose risks to the safety of human life during these survey activities. Consequently, due to these issues, only a small percentage of the ocean floor has been mapped to date [15].

Fortunately UUV are becoming more and more efficient in marine exploration activities and can therefore solve some of the problems highlighted [16]. In order to carry out exploration and survey activities of submerged environments, the use of appropriate subaqueous vehicle position estimation techniques is essential. Only by knowing the position of the vehicle and its sensors is it possible to safely carry out a survey and produce results that can be compared with other surveys and georeferenced in maps and DT. Increasingly precise instruments and sensors allow for accurate positioning and advanced navigation techniques now allow efficient use of UUVs for surveying [17].

In order to study in depth the complex underwater environment and map different habitats, survey methodologies from different platforms with optical and acoustic sensors have been successfully experimented in the past [18,19,20]. Due to the complexity of the underwater environment, UUVs are the only instruments capable of investigating the hostile underwater environment in some particular contexts. Several authors have approached the problem of controlling UUV vehicles with different methods to stabilize their attitude during navigation [21,22,23,24]. UUVs are particularly critical when observations must necessarily be made with high spatial as well as temporal resolution [25]. For example, using acoustic techniques such as Multibeam Echosounder (MBES) [20] it is already possible to survey high-resolution environmental data to accurately map and provide an automatic unsupervised or supervised classification of benthic communities without the use of invasive techniques [18,26].

Hopefully, thanks to ongoing technological and knowledge advances, UUVs could already in the next decade reach a level of reliability, performance, efficiency and economy of use similar to that which today belongs to the UAV domain: extremely versatile and economical to produce maps and digital models of numerous contexts related to the marine environment [27,28,29,30,31,32]. Moreover, it will be very useful to be able to draw up a list of strategies and best practices for underwater monitoring as is already happening in the terrestrial field [33].

In this paper we describe the challenges and lessons learned from the multipurpose UUV prototype, expanding on the work published in [34], developed within the Interreg Italy–Croatia SUSHI DROP Project (SUstainable fiSHeries wIth DROnes data Processing) https://www.italy-croatia.eu/sushidrop (accessed on 15 January 2022). The objective of the project is the complete development of a UUV for surveying the benthic zone in the deep waters of the Adriatic Sea that is able to produce digital models of the underwater environment.

We had the opportunity to develop a UUV prototype from scratch that combined the best possible features in terms of positioning and surveying while maintaining a low total cost and with multipurpose and modularity features. The developed prototype is innovative considering the entirety of its capabilities and the design choices that have identified operational solutions for the project objectives. The result of this work is a platform suitable for gathering information and providing efficient environmental investigation using non-invasive techniques in remote underwater environments. As UUVs are by definition unmanned, they also eliminate the risk of human operators and are therefore suitable vehicles for the exploration of a particularly hostile environment such as the marine one.

Until today, the principal instrument to collect information about fish populations and communities is from the analysis of catch collected during surveys performed with research vessels. Traditional survey methods that rely on the capture of living organisms are employed aboard these vessels. Capture is typically accomplished through the use of bottom trawl gear. These traditional methods are invasive to the environment, have high operational costs and are also prohibited in particular marine protected areas. The captured fish is then manually sorted by a group of specially trained operators. For each species, a record is made of the biological variables: length, weight and other key characteristics. This information is useful to understand the specificities of marine populations in the area under investigation. To date, only in some monitoring applications can be found that the data collection phase is instrumentally automated through the use of computer databases at the end of the manual selection process [35].

## 2. Materials and Methods

The approach followed for the development of the UUV was to produce a complete and modular prototype system, called Blucy, fully customized in hardware and software for scientific surveys, instead of using a commercial drone with black-box operation.

**Figure 1:**
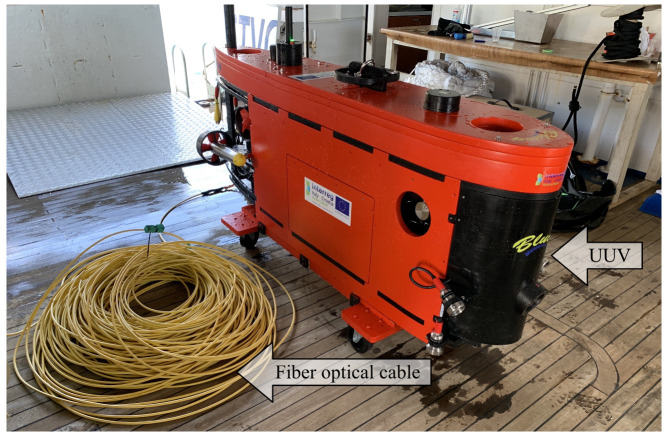
UUV prototype, Blucy and fiber optical cable during first tests in open sea.

Numerous marine robots have been developed in recent decades [36]. In general, UUVs can be divided into the following categories: Remotely Operated Vehicles (ROV) [37,38], Autonomous Underwater Vehicles (AUV) [39,40] or hybrids [41]. The new Blucy prototype (Figure 1) falls into the latter category, according to an approach that has made it possible to develop a drone at a considerably lower cost than comparable commercial vehicles.

This choice is dictated by the desire to maintain a dual option of autonomous navigation, but at the same time the ability to transmit high-resolution images, video stream, acoustic measurements and any other data of interest in real time. This need is particularly useful for the study of living organisms. The ROV mode is particularly useful for missions in complex areas, while the AUV mode carried out in complete autonomy allows to obtain information on large area such as the morphology of the seabed to be investigated in further detail.

### 2.1. UUV Payload

All subsystems within the UUV have specific operational capabilities dedicated to piloting, positioning or surveying. This paper is not focused on the piloting part, but is instead intended to deepen the challenges related to positioning and surveying techniques for UUV [42], explaining the tools and methods used and their specific implementation in Blucy.

A first precaution necessary for the proper functioning of all subsystems is a precise synchronization of the clocks of the different sensors: fundamental for the correct alignment of the acquired data and to guarantee a high quality of the following processing. All data are synchronized using UTC timestamps and are then georeferenced thanks to the coordinates obtained by the positioning instruments.

A second necessary precaution is the choice of subsystems. The approach followed had to consider the small size of the sensors to allow installation in the limited space available within the UUV. At the same time, among the sensors available for use on the UUV platform, a further parameter of choice was the analysis of power consumption that must necessarily be contained to ensure greater autonomy. Finally, the price–performance ratio was also considered with the aim of keeping costs low.

#### 2.1.1. Positioning Instruments

A multitude of dedicated positioning tools are provided on board the UUV, starting with the global positioning system that allows absolute localization of the drone during surface navigation. Internally to the Attitude and Heading Reference System (AHRS) instrument are a Global Navigation Satellite System (GNSS) receiver and an Inertial Navigation System (INS) platform. Additional instruments provided for relative positioning during submarine activities are: Fiber Optic Gyroscope (FOG), Doppler Velocity Log (DVL) and Ultra-Short Baseline (USBL). The information acquired by the instruments is processed in real time by Extended Kalman Filter (EKF) according to a Fossen model [43]. These positioning systems are used in real time for navigation operations and thus for piloting Blucy. They are also essential during data processing to provide an accurate georeferencing of submarine surveys and return of project outputs in a universal reference system.

The data acquired by sensors such as MiniCT and MiniSVS are also essential for precise positioning: conductivity, temperature, pressure and speed of sound. These sensors are used to improve the depth estimation of the UUV. In addition, the data obtained from MiniSVS are used for sound velocity correction within the algorithm for USBL. The same data are crucial for MBES during beam forming and beam steering.

**Figure 2:**
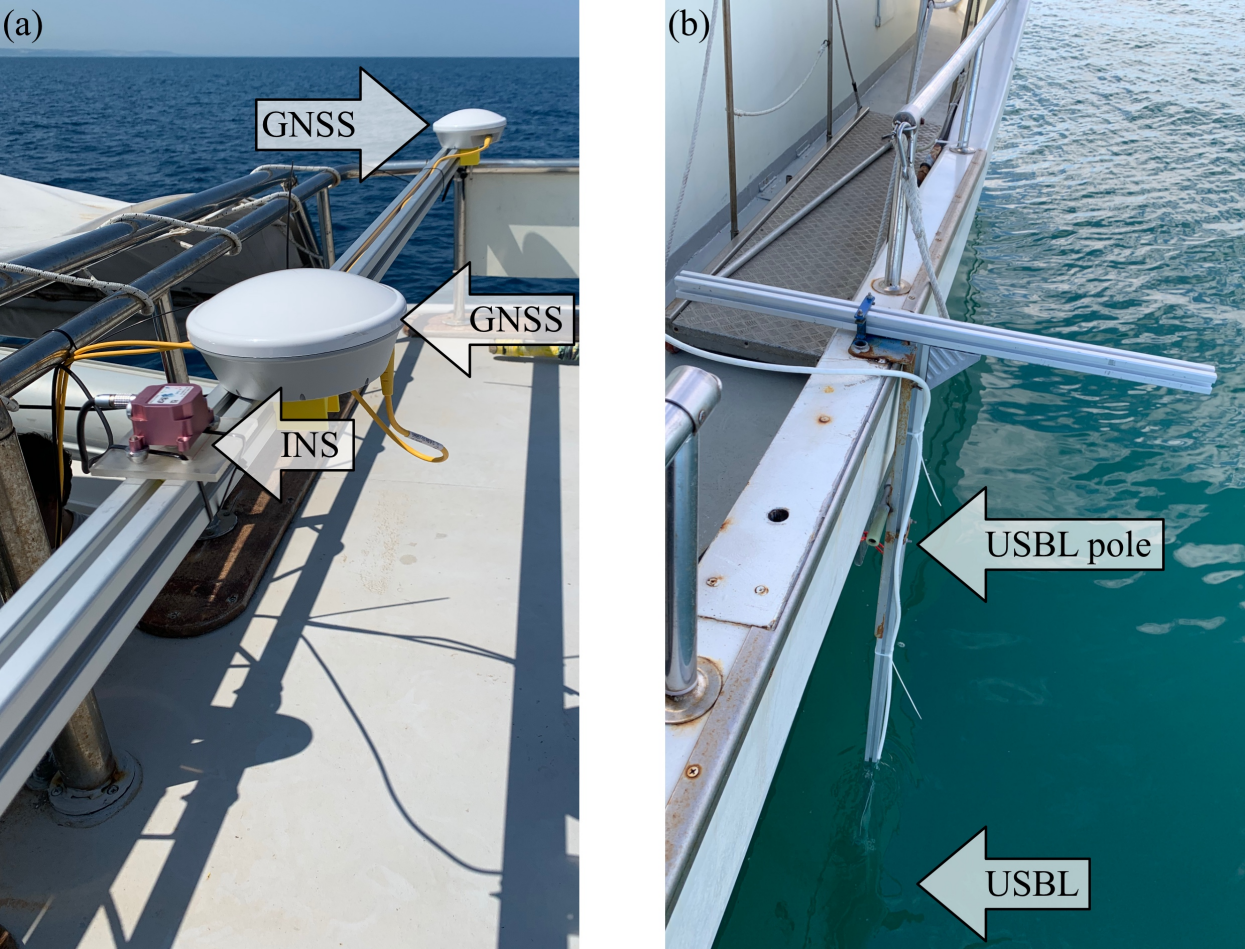
Setup for USBL positioning on surface vessel: (**a**) GNSS antennas and INS platform. (**b**) Structure for USBL.

Positioning instruments are used at different stages during the mission: the start of operations occurs above the water surface with the ability to acquire positioning using AHRS by identifying the position and attitude of the UUV in a geographic reference system. In the underwater navigation phase the GNSS signal is no longer acquired and the positioning is acquired in dead-reckoning using the mentioned procedure via EKF with data detected in combination with FOG, DVL and USBL. For the latter system, it is crucial to implement a correct reference on the surface vehicle by accurately measuring the offsets between the instruments on the boat. In the installation foreseen for the project operational scenario, two GNSS antennas are present to obtain an absolute positioning of the surface vessel and a heading angle, while the pitch and roll parameters are measured by an additional INS platform properly positioned and calibrated. The 3D offset (X, Y, Z) and rotation (rX, rY, rZ) between the GNSS surface vessel Antenna Reference Point (ARP) and the USBL transponder ARP, bound to the structure but appropriately positioned underwater (Figure 2), is measured on the boat and the resulting 3D vector is reported in the Remote Station (RS) software dedicated to USBL positioning.

Using these positioning techniques it is possible to check in real time the position of the UUV during underwater navigation and to know its attitude. Positioning information is transmitted by cable in ROV mode and by acoustic channel in AUV mode. Thanks to the on-board instruments it is also possible to obtain redundancy on the position estimation.

Positioning instruments are listed in Table 1. Regarding the choice of positioning tools, the first selection concerned the FOG platform, which is the main element for dead-reckoning navigation. Further components have been selected to ensure maximum compatibility and interoperability between the different subsystems.

#### 2.1.2. Surveying Instruments

**Figure 3:**
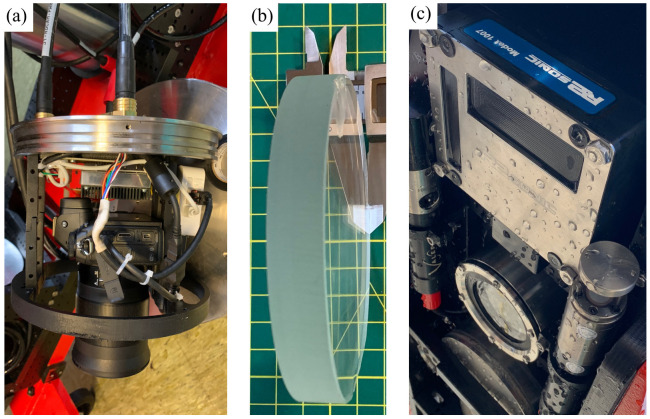
Main surveying instruments: (**a**) BottomCAM and its network data transmission router before insertion into the waterproof case. (**b**) BottomCAM 15 mm frontal glass. (**c**) MBES sonar head (top) and final BottomCAM canister (bottom) installed in UUV.

A number of scientific instruments have been provided on board the drone for multi-parameter remote sensing survey of the marine environment. These include several passive and active sensors. In the current configuration there are two optical cameras, PilotCAM and BottomCAM, and an MBES (Figure 3).

The first optical camera is positioned in the frontal part of the drone and the sensor coupled to its lens has been selected to obtain an image with wide Field of View (FOV). The typical output of this sensor is a video stream transmitted in real time thanks to the wide bandwidth available in ROV mode using the fiber optic cable that connects the UUV with the RS. The sequence of images immediately visible on the RS allows precision navigation at particularly close range to the seabed. The same images are used for inspection, visual census and the video stream can be reprocessed afterwards with different Computer Vision algorithms. PilotCAM is installed on a special custom support that allows us to change the inclination angle of the sensor according to the different operational scenarios of the mission.

BottomCAM is installed at the bottom of the UUV with viewpoint towards the seabed (nadir) to allow use for photogrammetric purposes. Thanks to the high resolution images and to the presence of several LED illuminators it is possible to acquire sequences of images following a specific survey plan. Parameters such as overlap, shutter speed are then studied and the height above the seabed is selected according to the necessary Ground Sample Distance (GSD) and visibility conditions in the water column. The optics selected for the setup has a focal length of 24 mm, ensuring a wide FOV. Following a classical photogrammetric scheme, the UUV navigation in the BottomCAM survey operations will follow a path maintaining constant altitude to avoid distortions in the final model. In ROV mode the shooting command is automatically given by RS according to the survey parameters. The bidirectional communication takes place according to network protocols through the fiber optic cable. Inside the waterproof container where the BottomCAM is located it has been foreseen the installation of a signal conversion module suitable to remotely control the camera and receive in real time the images acquired by the UUV on the RS. This allows an immediate processing using a particularly high performance workstation.

The MBES active acoustic sensor is used to detect information and produce maps related to more distant objects. It is possible to survey the seabed, acquire high quality bathymetric data, water column information and quantitative fisheries stock assessment for habitat mapping, which is one of the main goals of SUSHI DROP Project. MBES can be used in both ROV and AUV modes. In the second mode all acquired information is stored in a dedicated on-board computer present inside the UUV architecture. In this case the data are only subsequently downloaded to the RS at the completion of the mission, due to the reduced bandwidth available for acoustic communications. Other parameters concerning the water column are punctually acquired during the navigation by MiniCT and MiniSVS sensors: conductivity, temperature and sound velocity.

It is necessary to consider some parameters that generate problems and distortions in the acquired images including the marine housing where the optical sensor is kept watertight and the additional glass that is interposed between the sensor and the external environment. Algorithms are needed to correct the refraction according to the different mediums (air–glass–water) that are crossed by the optical rays [44].

All surveying instruments equipped on the UUV are listed in Table 2.

### 2.2. UUV Architecture

The multi-purpose UUV developed for the project and named Blucy, is designed to operate in a dual hybrid navigation mode: ROV or AUV depending on the mission setup. In both cases, power is supplied to the UUV by an integrated battery, with no need for remote power transmission. The UUV is built for depths up to 250 m.

**Figure 4:**
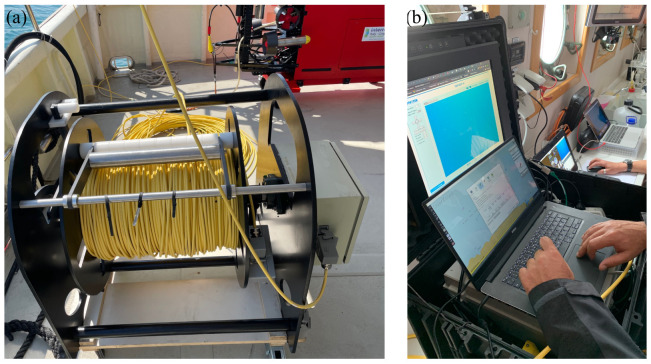
ROV navigation mode: (**a**) 600 m long fiber optic cable. (**b**) Computers for RS on surface vessel.

In ROV navigation mode, Blucy is tethered to the surface ship with the help of a 600 m long fiber optic cable (Figure 4). The cable does not carry energy as this is supplied directly on board the drone to allow operation in dual mode. The selected fiber optic cable has a nominal overall diameter of 7.80 mm, specific gravity of 0.95 kg/dm^3^, LCP fiber braid strength member, LDPE UV resistant sheath, hydrolysis UV resistant PUR outer sheath and a total breaking strength of 500 kg. The ROV mode is mainly used for close inspection of the seabed performed in hostile environments and also in particularly tight spaces at moderate cruising speed and when high position accuracy is required. In this mode all data are transmitted in real time and recorded on the RS rugged computer.

The AUV navigation mode is suitable for surveying large areas at high cruising speed in safe conditions, considering that the maximum range of Blucy is 6 h of continuous navigation. A typical survey in AUV mode must necessarily follow a path previously planned through the use of way-points at a safe distance from the seabed and any other obstacle. Furthermore, to ensure the necessary performances and increase the reliability of Blucy during the AUV missions, a supervision scheme for early actuator Fault Detection and Isolation (FDI) is required [45,46]. With the scientific sensors equipped on board, including MBES, it is possible to analyze elements more than 100 m away from the AUV position while maintaining an adequate resolution for the study of the seabed. In this mode the status of the AUV, its position and commands are received and transmitted with a dedicated acoustic channel via USBL, connecting the AUV to the RS present on the surface vessel. The underwater acoustic modem used provides a full-duplex digital communication, using self-adaptive algorithms to maintain a nominal bitrate up to 13.9 kbit/s with a bit error rate less than 10${}^{-10}$, depending on marine conditions. Among other challenges to face for acoustic communication and submarine digital data transmission, we can mention some that also concern the AUV sector: attenuation, time synchronization, data transmission delay, underwater noises, stratification and multi-path effect [47]. These problems affect the ability to control UUV navigation in real time. At this stage of development the large amount of data acquired by the drone cannot be transmitted in real time in AUV mode. All data are recorded on a solid-state drive (SSD) within dedicated subsystems and then downloaded at the end of the mission.

**Figure 5:**
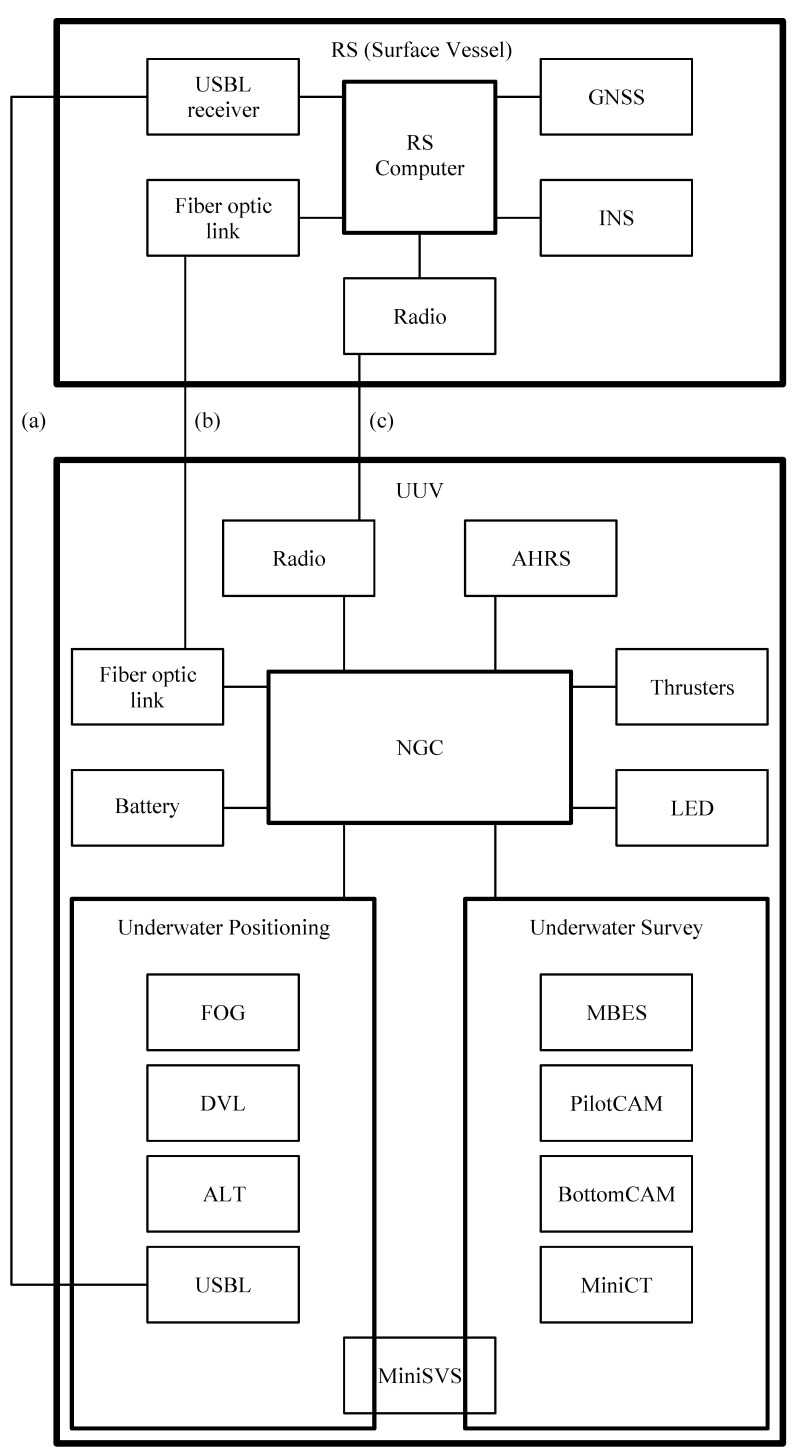
UUV main functional architecture and connections: (**a**) Acoustic USBL channel for AUV underwater navigation. (**b**) Fiber optic cable for ROV mode. (**c**) Radio link for AUV surface navigation and data download.

All UUV subsystems and their functional connections are listed in Figure 5.

### 2.3. Survey Methods

The activities foreseen for each mission at sea are carefully prepared before the activities following a strict checklist of sequential operations that can be divided into a few main phases described below in the fundamental elements:

1. Dry Calibration for Navigation and Positioning;
2. Wet Calibration tests for Navigation and Positioning;
3. Wet Calibration tests for Scientific Survey;
4. Mission Planned Survey.

#### 2.3.1. Dry Calibration for Navigation and Positioning

During this phase, all the navigation and positioning systems on board Blucy are initialized. In particular, the AHRS fixes the GNSS position signal and initializes the FOG for position estimation during dead-reckoning.

#### 2.3.2. Wet Calibration for Navigation and Positioning

At this point, Blucy is deployed from surface vessel and a series of calibration maneuvers with the vehicle on the surface were performed. In detail, maneuvers are carried out by piloting Blucy both in manual mode and with autopilots engaged to verify the correct functioning of the navigation guidance and control subsystem and the propulsion system. If the navigation performances are not satisfactory, fine-tuning of the autopilots parameters can be performed directly from the ground station. Moreover, during this phase, the buoyancy check of the drone is performed. This last operation must always be carried out before the mission because the salinity conditions or the presence of freshwater significantly modify the buoyancy characteristics. During UUV development phase, the option of integrating a self-adaptive buoyancy system adding an additional subsystem to act as Buoyancy Control Device (BCD). This design choice would have increased the complexity of the UUV by adding a subsystem subject to potential failure. In particular, since BCD is an active system, the issues of a failure are critical and could lead to a potential loss of UUV during underwater missions. Finally, with the same final UUV size, a bulky subsystem such as BCD would have subtracted space for scientific payload.

#### 2.3.3. Wet Calibration for Scientific Survey

During this step, the scientific sensors are calibrated, in particular a vertical survey is carried out to know the seabed depth and to characterize the water column in terms of sound speed, conductivity and temperature. These physical quantities will be used both for sensors calibrations and for further scientific post processing analysis. Moreover, based on the seabed depth, Blucy hypothetical navigation altitude and physical data previously gathered, MBES mission profile is determined. The MBES mission profile allows us to automatically change power, pulse width and gain based on the range settings, allowing an automatization of MBES data acquisition routines. The choice of these parameters is fundamental since a wrong setup could compromise the quality of the gathered data, increasing the post-processing time of those data or in the worst case making them meaningless.

### 2.4. Mission Survey

The surveys are distinguished by the main scientific payload used and the size of the area to be surveyed. Typically the following two operations are performed:

1. MBES Survey;
2. Close Seabed Survey.

**Figure 6:**
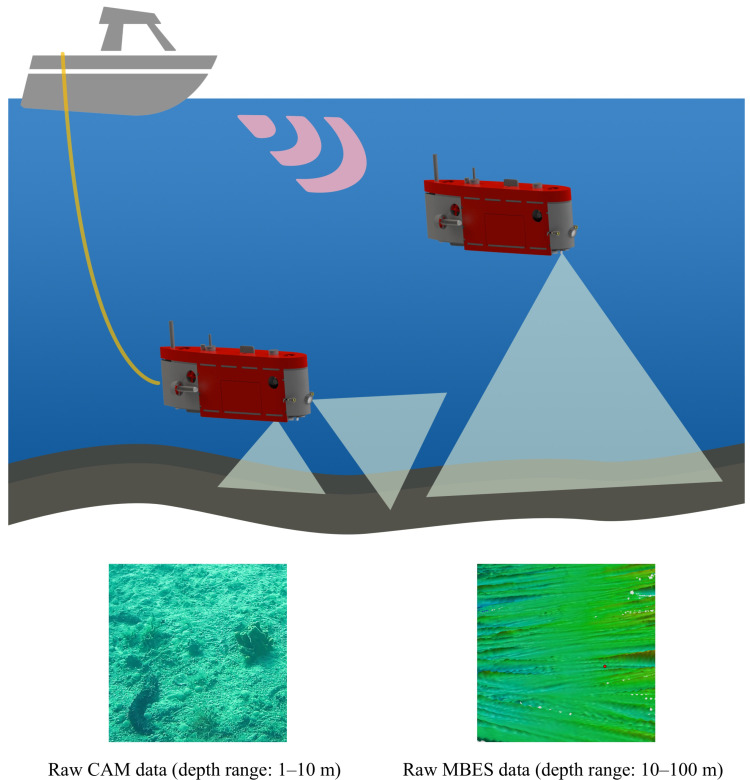
Graphical comparison between MBES in AUV mode and close seabed survey with BottomCAM and PilotCAM in ROV mode.

It has to be noticed that the two types of surveys differ in the type of data acquired, time of acquisition in terms of area surveyed, Blucy navigation mode and 3D waypoints or survey lines (Figure 6).

#### 2.4.1. MBES Survey

Using MBES, it is possible to scan a large area [48] in a short time. At this stage Blucy can be piloted in both navigation modes, ROV or AUV, because it navigates at constant depth and is not affected by surface currents or wave motion. At the same time it is always at a safe distance, away from the seabed, avoiding any interference with it. The 3D reconstruction resulting from these acquisition could allow to identify hot-spots, areas of limited extension, in which perform further close seabed inspection using optical sensors, increasing the level of detail and knowledge of the area surveyed. It is important to highlight that the MBES is independent of the visibility conditions of the water and relative turbidity.

The following factors were considered when planning a survey with the MBES:

- The geography and extension of the survey area;
- Suitable areas for calibration patch test;
- Echo sounder coverage;
- Seabed topography;
- Sound Speed variations;
- Weather conditions.

In MBES survey, the operator has to plan the surveys lines carefully based on swath coverage that defines the multibeam system. The survey lines should be designed so that there is at least 80% overlap in coverage between adjacent lines. As swath width is a function of distance from the seafloor, it follows that the spacing between the lines may not be constant. The planning survey lines are designed with a trade off between Blucy navigation depth, seabed morphology (slopes, rocky formations), seabed type (grain size characteristics of the seabed) and swath width. With MBES the lines are planned to be perpendicular to slope directions to maintain a constant swath coverage. Even if navigation takes place at a depth for which surface currents can be considered negligible and maintaining an attitude aligned to the seabed with zero pitch and roll, it is still useful to calibrate the offsets regarding MBES according to the following procedure:

- Roll: two lines over a flat area in opposite direction with the same speed;
- Pitch: two lines over an area with slopes (or an object) in opposite directions with same speed;
- Heading: two lines over an area with slopes (or an object), the lines need to overlap half a swath width, in same direction with same speed.

#### 2.4.2. Close Seabed Survey

As above mentioned, starting from the MBES 3D reconstructions, hotspots can be identified that can be inspected by optical sensor to obtain deeper morphological data. During this operation it is necessary to use Blucy in ROV mode because it navigates in hostile environment with low altitude from 5.0 m to 1.0 m depending on turbidity found in surveyed area. Furthermore, the data are transferred in real time with fiber optical cable with constant supervision by an operator who can perform corrective navigation maneuvers as needed. Moreover, the acquisition of appropriate optical images is possible only in situations of suitable seabed with a reduced suspension of sediment in water, at close distance from the seabed itself and in particular periods of the year in view of the fixed marine currents [49]. In spite of the previous precautions, it may also be necessary to improve the images during post-processing through the use of specific algorithms [50] and color correction [51].

In this operation the survey lines are designed taking into account the following parameters:

1. Constant Blucy navigation altitude from the seabed based on water turbidity;
2. FOV at seabed;
3. Speed of navigation;
4. Interval between images;
5. Appropriate overlap for underwater surveys [52].

Using the 24 mm focal length of the BottomCAM lens at a reference altitude of 5.0 m, it is possible to cover a seabed swath width of 7.5 m with a GSD of 1 mm, thanks to the 24 mpixel sensor.

## 3. Results and Discussion

### 3.1. Prototype UUV

**Figure 7:**
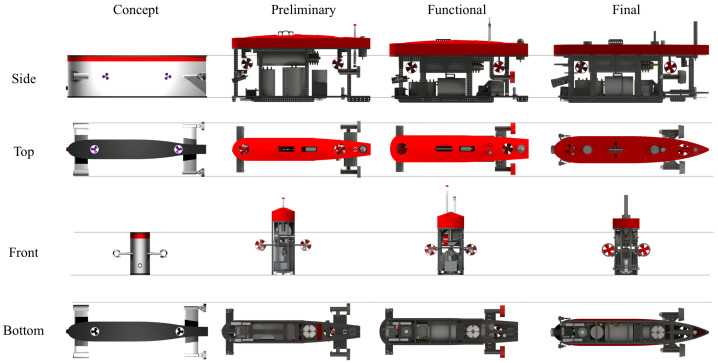
Four stages of design for Blucy UUV.

One of the main results of the SUSHI DROP Project is the realization of the complete prototype UUV previously designed and studied in the different components: sensors and subsystems. With the scientific payload available, procedures were tested to survey the data necessary to create a marine DT. The development of the drone took place following an iterative process, starting from the theoretical specifications and verifying the specifications of the components available on the market to select the different subsystems. The goal was to create a hybrid shape optimized for both high and low cruising speeds with a functional set-up for both ROV and AUV modes. ROVs often turn out to be box-shaped, while AUVs are torpedo-shaped. Blucy initial design was then iteratively modified in four stages of design: concept, preliminary, functional and final (Figure 7). The final UUV has an approximate weight of 200 kg including the on-board battery and the following main body dimensions in millimeters: Length 2000, Width 350, Height 740.

The final shape of the prototype UUV allows for a reduced roll during navigation and particularly during survey activities, compared to typical AUVs with a torpedo structure. This design choice improves both the navigation ability allowing a better NGC, and the possibility to perform a better optical and acoustic survey minimizing the need to compensate for the attitude of the vehicle.

Each element of the Blucy scientific payload was carefully positioned to maximize its effectiveness during survey activities. The positioning and communication sensors are located at the top of the drone, above the buoyancy foam layer, to avoid any interference. In the barycentric part of the structure there is the on-board computer and under it the heaviest element: the battery. All survey sensors are located in the lower part and are coupled with LED illuminators. The PilotCAM is mounted at an angle of about 45 degrees with respect to the seabed, while the photogrammetric BottomCAM is mounted perpendicularly to allow a three-dimensional reconstruction and the realization of the Digital Elevation Model (DEM) and orthomosaics of the seabed. The MBES is also positioned at the front with a tilting head, while FOG, DVL and altimeter sensors are at the rear. All on-board acoustic instruments were carefully selected with appropriate technical specifications to avoid any interference during mission activities. The different subsystems operate in different operating frequency bands: USBL 18–34 kHz, ALT 200 kHz, MBES 200–450 kHz and DVL 500 kHz.

Regarding the propulsion the UUV is equipped with a pair of thrusters on each axis: two vertical, two longitudinal and two lateral for a total of six thrusters.

**Figure 8:**
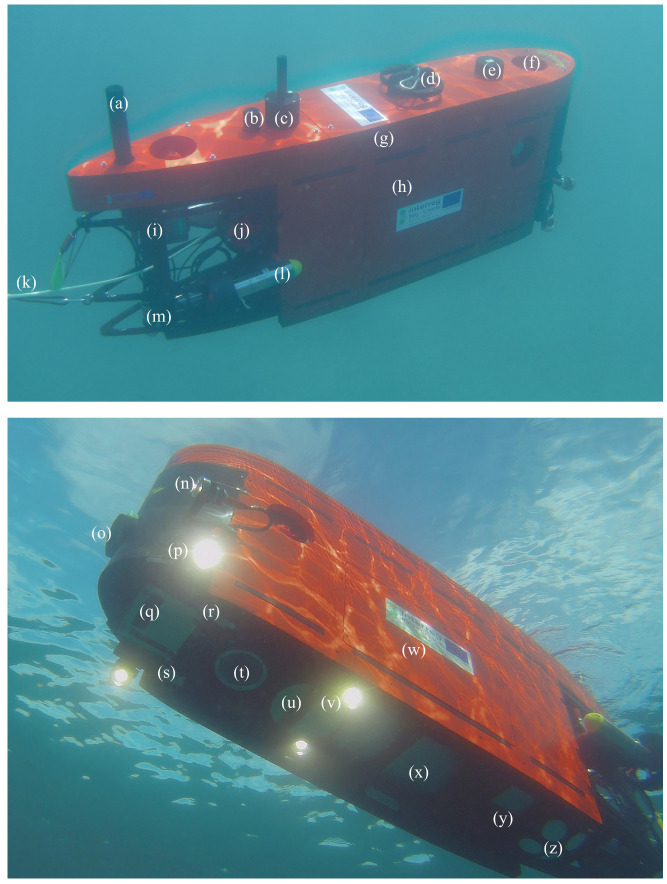
Main prototype UUV components: (**a**) Wi-Fi communication. (**b**) USBL transponder. (**c**) Radio communication. (**d**) Hook. (**e**) AHRS: GNSS and INS. (**f**) Thruster: vertical. (**g**) Main buoyancy foam. (**h**) Lateral buoyancy foam panel. (**i**) HDPE structure. (**j**) Thruster: lateral. (**k**) Fiber optic cable. (**l**) Thruster: longitudinal. (**m**) Altimeter. (**n**) Frontal LED lights. (**o**) PilotCAM. (**p**) Adjustable LED lights. (**q**) MBES sonar head. (**r**) MiniSVS. (**s**) MiniCT. (**t**) BottomCAM. (**u**) MBES computer. (**v**) Bottom LED lights. (**w**) Navigation, Guidance and Control (NGC) computer. (**x**) UUV 24V Battery. (**y**) FOG. (**z**) DVL.

UUV components are listed in Figure 8.

**Figure 9:**
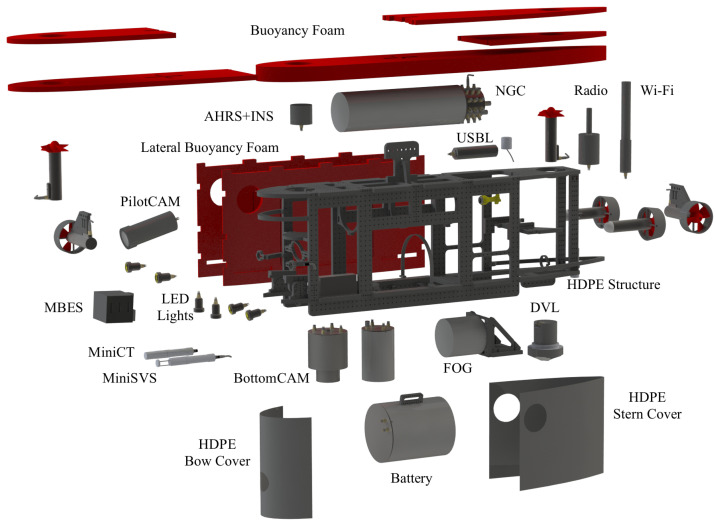
Exploded view technical drawing of Blucy UUV structure and hardware subsystems.

The features of the designed drone allow it to be multipurpose and modular. Being able to rely on a precision navigation for detailed inspections and at the same time a propulsion sufficient to cover large areas it is possible to perform different operational scenarios within the same mission: MBES survey and close seabed survey. Finally, thanks to the modularity of the architecture, it is possible to operate in two complementary modes (ROV or AUV) with the additional possibility of exchanging the scientific payload and possibly each secondary subsystem according to mission requirements (Figure 9).

### 3.2. UUV Deployment

**Figure 10:**
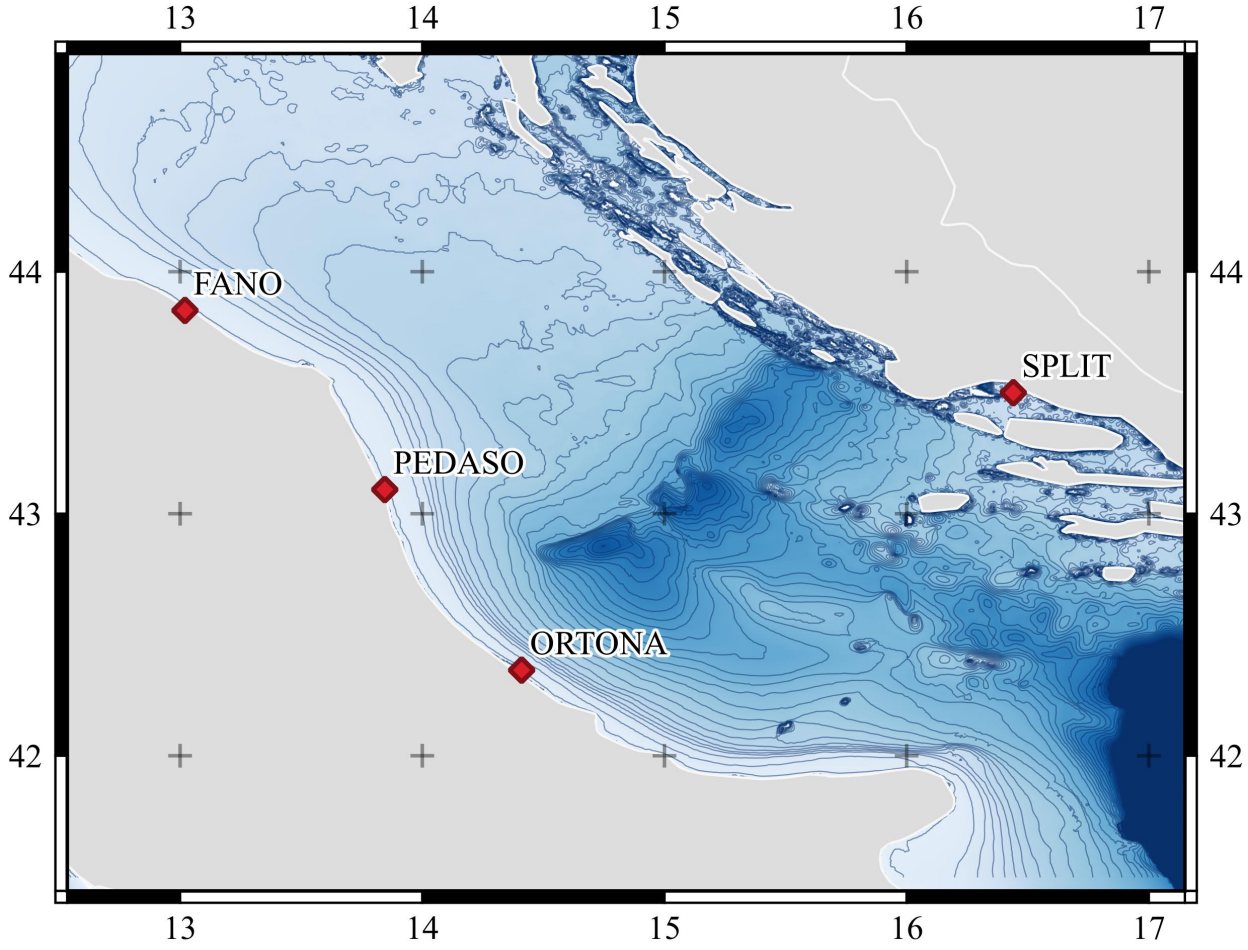
Area of SUSHI DROP marine missions. Coordinates WGS84, bathymetric lines at 10 m interval from GEBCO Bathymetric Compilation Group 2021 (2021). The GEBCO 2021 Grid—a continuous terrain model of the global oceans and land. NERC EDS British Oceanographic Data Centre NOC https://doi.org/10/gn6h (accessed on 15 January 2022).

The sea missions under the SUSHI DROP project took place in the year 2021 near the Italian and Croatian coast. The sites covered by the mission in Italian territory include the areas near the town of Fano, Pedaso and Ortona in the Costa dei Trabocchi. In Croatian territory several missions were carried out in the portion of the sea in the waters of Split. With a wide selection of case studies it was possible to evaluate different characteristics of biodiversity by sampling significant areas in the Adriatic Sea basin. The areas where project missions were carried out are shown in the Figure 10.

**Figure 11:**
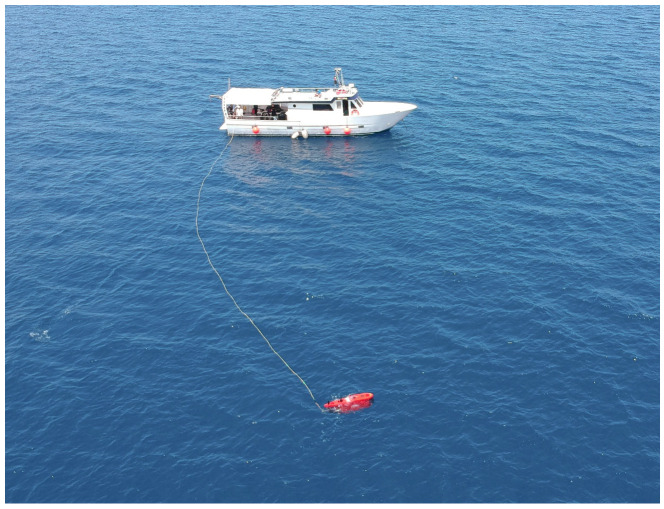
Blucy deployment from surface vessel in ROV mode during SUSHI DROP project mission.

During in-water activities, it was critical to consider the conditions of the different mission sites in terms of currents and water turbidity. In particular, when the UUVs are configured in ROV mode (Figure 11) for close seabed survey, they are affected by the presence of strong currents also due to the presence of the cable and related management challenges [53].

The unambiguous use of a geographic reference system was essential for the missions performed, both during navigation and data processing. In fact, only through the use of a correct coordinate system is it possible to ensure the repeatability of the survey, generating maps and models as DT accompanied by the necessary metadata for the correct scale and georeferencing of spatial information.

**Figure 12:**
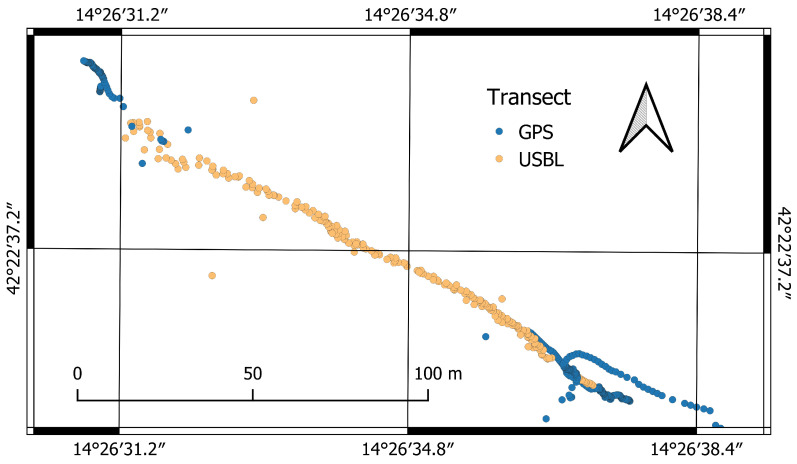
Transect performed by UUV partially above and partially under water surface.

The result provided in Figure 12 is an example of a transect performed by the UUV, partially above sea water and partially submerged, shows the effectiveness of the positioning sensors after rigorous calibration. The sequence starts from the southeast with the drone on the surface positioned by the on-board GPS receiver shown by the points highlighted on the map with blue color. When the drone submerges, it can no longer receive the satellite signal and relative positioning is activated via USBL, respectively, to the surface research vessel, shown on the map with yellow points. The drone performs the optical and acoustic survey remaining submerged and at the end of the transect, when it re-emerges in the north-west part of the image. There is a substantial coincidence of positioning, excluding some outliers especially at the air–water interface, where neither GNSS signals nor acoustic ones are stably received. The map shows a reduced drift of positioning and allows an accurate georeferencing of the acquired data.

Regarding submarine navigation with the absence of fiber optic cable, data transmission is particularly low in bandwidth and subject to the critical issues described in Section 2.2. The architecture of the UUV ensures sufficient bandwidth to transmit the acquired data when the vehicle is on the water surface through the Wi-Fi antenna. At this stage of development further testing needs to be performed to optimize the data transmission during underwater acquisition through the USBL acoustic channel. Future developments, which go beyond the topics discussed in this paper, will involve the application of signal compression algorithms to minimize the size of the acquired data and allow it to be transmitted over the acoustic channel.

### 3.3. Surveyed Habitats

#### 3.3.1. Marine Biodiversity

**Figure 13:**
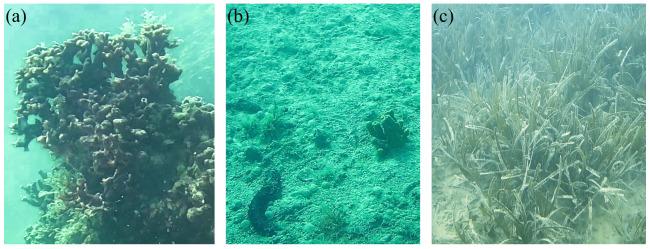
Some marine species identified in images acquired by PilotCAM: (**a**) *Schizoporella errata*. (**b**) *Axinella* sp., *Holothuria* sp., eggs masses of *Polychaeta*. (**c**) *Posidonia oceanica*.

Thanks to the use of the raw data obtained from Blucy’s optical sensors, it was possible to conduct a qualitative study, assess the presence or absence of marine species characteristic of an inspected ecosystem during the first surveys. Visual census analysis consists of the identification and counting of species (e.g., fishes, benthic species) observed within a defined area. Visual census can be used to estimate the variety, numbers and even sizes of common, easily seen, easily identified species in areas where the recorded quality images were very good. A first preliminary video assessment analysis is performed in real-time during navigation to better plan the mission. More detailed work is carried out in the laboratory by processing all the photographic frames taken by the high-resolution BottomCam and the video stream recorded by the PilotCam. The strength of ROV-imaging is the ability to explore the seabed without resorting to the use of scuba divers. In addition, it is a non-invasive technique that allows the evaluation of the ecosystem without impacting on the benthic species present, unlike what happens with the classic sampling techniques that require the removal of the individual from its natural environment. Thanks to the images provided by the UUV it was possible to census numerous benthic species such as: holothurian, sponges, hermit crab, cnidaria and Posidonia as represented in (Figure 13).

**Figure 14:**
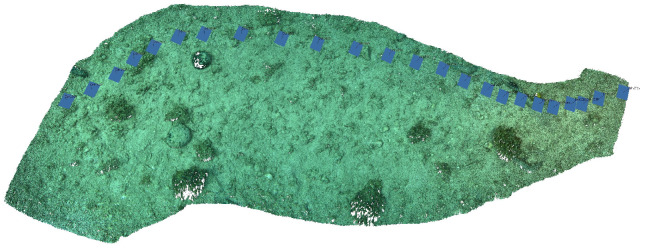
Dense 3D point cloud of seabed processed from BottomCAM high-resolution imagery with SFM techniques.

In our survey we focused on the seagrass meadow (e.g., Posidonia oceanica). Due to its ecological role is an EU priority habitat, it is provided important ecosystem services: they contribute to coastal primary production and nutrient cycling, providing food, shelter, nurseries and habitat for many vertebrates and invertebrate species. The shift from qualitative studies, as described above, to quantitative studies requires a data processing phase. By using telemetry information collected simultaneously by the subsystems on Blucy, it is possible to georeference large portions of the acquired images and produce metric products such as orthophotos of the seabed. Furthermore, exploiting Structure from Motion (SFM) from imagery or MBES data processing techniques, it is possible to obtain a three-dimensional reconstruction of the marine environment and an high-resolution DEM of the seabed (Figure 14).

#### 3.3.2. Mussel Farming

**Figure 15:**
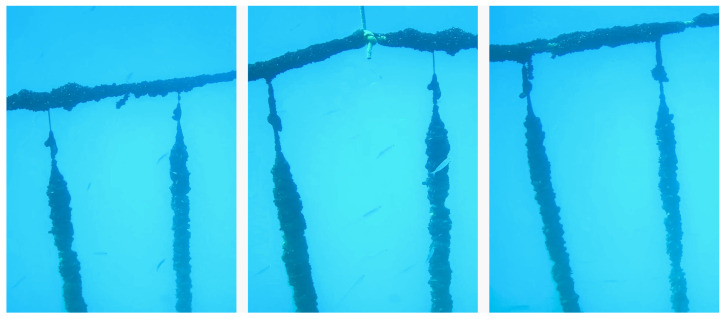
PilotCAM image sequence for mussel farming nets: Mitylus galloprovincialis.

Worldwide production of consumer fish products is 45 percent derived from aquaculture farms [54]. This requires all infrastructure and equipment to function properly, ensuring compliance with health and hygiene standards, ensuring the integrity of seafood farms can be a delicate challenge. A UUV such as Blucy can be used in an innovative way providing aquaculture farms with durable, easy to use and affordable underwater inspection and inspection systems for daily operation and maintenance. In a field mission Blucy was used, in a completely non-invasive way, in a mussel farm (Figure 15). Specifically, the daily maintenance of mussel farms requires a constant use of boats and crew in order to meticulously inspect all rows/poles of the farm. Thanks to the instrumentation on Blucy, it is possible to acquire data on the health and growth of the mussels such as consequent reduction in the daily use of boats. This approach would lead to a drastic reduction in the costs required for the boat and its crew, as well as significantly reducing the environmental impact of maintenance operations. Using the data collected by MBES and optical analyses, it is possible to estimate the health status of socks. This approach is already used in precision agriculture, where by using 3D reconstructions it is possible to define the state of growth by volumetric analysis. Moreover, during the survey, Blucy has the possibility to record biochemical properties of the water column. All the data gathered by the UUV, in addition to meteomarine information, are the ideal input for the design of a predictive model of the mussel growth status, leading to a further optimization of subsequent missions and a potential reduction in anthropogenic actions.

## 4. Conclusions

As part of the Interreg project SUSHI DROP, a working prototype of a multi-purpose UUV has been developed and equipped with a multitude of selected instruments to non-invasively investigate the marine environment and produce population estimates of fish stocks. On-board sensors enable the acquisition of high-resolution optical and acoustic data while simultaneously monitoring physical, chemical and biological characteristics with precision. The prototype, called Blucy, is built with the possibility to operate in hybrid mode ROV or AUV. Among the various challenges for the realization of DT and accurate surveys of the seabed, until now largely unmapped, emerges the need for appropriate use of positioning techniques of the UUV and its sensors. Only through an accurate underwater positioning and a correct parameterization of the acquired information it will be possible to produce correctly georeferenced results and therefore comparable with other acquired data or subsequently replicable over time. Lessons learned as part of the SUSHI DROP project will contribute to the future deployment of a larger fleet of UUVs that will provide the scalability necessary to address the observation of critical habitats throughout the Adriatic Basin moving the first steps for the realization of a complete marine DT. In the near future, thanks to continuous technological innovations and scientific research, UUVs could achieve the same high level of efficiency, reliability and service economy that belongs to UAVs today.

## Figures and Tables

**Table 1:** Instruments for positioning of the UUV Blucy.

Sensor	Parameters	Model
FOG	Position, Attitude	iXblue Fiber Optic Gyroscope Compact C3
DVL	Linear Speed and Acceleration	Nortek Doppler Velocity Log DVL500 - 300 m
AHRS	Latitude, Longitude	LORD 3DM-GX5-45 GNSS/INS
ALT	Altitude	Tritech PA200
USBL	Relative Position	EvoLogics S2C M 18/34

**Table 2:** Instruments for scientific survey.

Sensor	Parameters	Model
MBES	Bathymetry, Water Column	Multibeam Echosounder R2Sonic 2020
PilotCAM	High Resolution Imagery	Nikon Z6 with NIKKOR Z 24 mm f/1.8 S
BottomCAM	Live Stream Video	Vivotek IB8369A Network Camera
MiniCT	Conductivity, Temperature	Valeport MiniCT
MiniSVS	Pressure, Sound Speed	Valeport MiniSVS

## Data Availability

Not applicable.

## Abbreviations

The following abbreviations are used in this manuscript:

AHRS	Attitude and Heading Reference System
AUV	Autonomous Underwater Vehicle
DEM	Digital Elevation Model
DT	Digital Twin
DVL	Doppler Velocity Log
EKF	Extended Kalman Filter
FOG	Fiber Optic Gyroscope
FOV	Field of View
GNSS	Global Navigation Satellite System
GSD	Ground Sample Distance
INS	Inertial Navigation System
MBES	Multibeam Echosounder
NGC	Navigation, Guidance and Control
ROV	Remotely Operated Vehicle
RS	Remote Station
SFM	Structure from Motion
USBL	Ultra-Short Baseline
UUV	Unmanned Underwater Vehicle
